# Silica/klucel nanocomposite as promising durable adsorbent for lead removal from industrial effluents

**DOI:** 10.1038/s41598-024-74680-2

**Published:** 2024-10-30

**Authors:** Shaymaa E. El-Shafey, Mohammed K. Obada, A. M. El-Shamy, Wael S. Mohamed

**Affiliations:** 1 Surface Chemistry and Catalysis Lab., Physical Chemistry Department, National Research Centre, El-Bohouth St. 33, Dokki, P.O. 12622, Giza, Egypt; 2 Egyptian Projects Operation and Maintenance Co. (EPROM), Petroleum Complex, Alexandria, Egypt; 3 Electrochemistry and Corrosion Lab., Physical Chemistry Department, National Research Centre, El-Bohouth St. 33, Dokki, P.O. 12622, Giza, Egypt; 4 Polymer Department National Research Centre, El-Bohouth St. 33, Dokki, P.O. 12622, Giza, Egypt

**Keywords:** Heavy metal removal, Silica/klucel nanocomposite, Lead adsorption, Industrial wastewater treatment, Eco-friendly adsorbent, Batch adsorption kinetics, Environmental sciences, Chemistry

## Abstract

The removal of heavy metals, such as lead, from industrial wastewater is imperative due to their detrimental effects on both human health and the environment. This study delves into investigating the feasibility of employing a novel adsorbent, specifically a silica/klucel nanocomposite, for effectively extract lead from industrial effluents. The synthesis of this nanocomposite involved a simple and cost-effective method, combining silica nanoparticles with klucel. XRD, FTIR, E-SEM, Raman, and N_2_ gas adsorption at − 196 °C tools were employed to prospect the formation of silica/klucel nanocomposite. Outstandingly, treating 50 ml of 50 mg/l of lead with 10 mg of adsorbent exhibited rapid removal, which reached a maximum (95%) at 60 min contact time. The resulting composite demonstrated remarkable adsorption capabilities, primarily attributed to two factors: the expansive surface area of silica nanoparticles 139.1 m^2^/g and the porous structure provided by klucel. Through batch adsorption experiments, the nanocomposite’s proficiency in removing lead ions from aqueous solutions became evident. The kinetics of the adsorption process were found to adhere closely to a pseudo-second-order model, hinting at chemical adsorption as the rate-determining step. Langmuir isotherm model revealed that lead ions tend to form a monolayer on the surface of the nanocomposite and the maximum adsorption capacity (q_m_) was 63.938 mg/g. Additionally, the nanocomposite, exhibited notable stability and could be reused multiple times, where 65% removal efficiency was announced until the 7th cycle without significant degradation in performance. In summary, the silica/klucel nanocomposite emerges as a promising and eco-friendly adsorbent for removing lead from industrial effluents. Its efficient performance and sustainability offer a compelling solution to combat heavy metal contamination, thereby contributing to environmental preservation and human well-being.

**Supplementary Information:**

The online version contains supplementary material available at 10.1038/s41598-024-74680-2.

## Introduction

Because of the increasing global population and our luxurious lifestyle, the requirement for high-quality water has increased gradually during the past few years^1^. Nevertheless, the extensive application of organic materials such as dyes and inorganic materials such as heavy metals, regarded as an illegal challenge to the water’s surface in addition to ground water, regularly tainted^2^. Heavy metals like lead are commonly found in industrial effluents, presenting significant risks to both human health and the environment due to their toxic properties and ability to persist in ecosystems^3^. Lead is notorious for causing severe health issues, including neurological disorders and cardiovascular diseases, even at low concentrations^4^. Industrial processes such as mining, metallurgy, electroplating, and battery manufacturing, as well as wastewater treatment plants, contribute to the widespread contamination of water sources with lead-containing effluents^5^. Therefore, it is crucial to remove lead from industrial wastewater to mitigate its harmful effects. Numerous techniques have been devised for the extraction of heavy metals, encompassing precipitation, ion exchange, membrane filtration, and adsorption, among others^6^. Among these methods, adsorption stands out for economic viability, speed, simplicity, and its acknowledged efficacy in heavy metal removal processes^7–9^. Numerous adsorbents, including activated carbon^10^, zeolites, bio-based materials, bi-functionalized materials, and mesoporous silica^11^, have been investigated for lead removal from aqueous solutions^12^. Silica nanoparticles, are known for their large surface area, porous structure, good recyclability, and excellent adsorption capacity. Silica was used separately or in composite form in a wide range for the removal of lead from waste water^5,11^. Several studies have shown that mesoporous silica showed improved adsorption capabilities for lead after treatment with organic and inorganic compounds such as OPW^13^, MoO_3_^14^ activated carbon^15^, FeNi_3_^16^. lignin biosorbent^17^ and Al_2_O_3_^18^.

Due to its organic nature, the presence of functional groups in its structure, and its low cost, hydroxypropyl cellulose polymer (klucel) can adsorb various ions, such lead (II)^19,20^. However, some disadvantages limit its use because of its low surface area and difficulty separating it^21^. Klucel disadvantages could be overcome by incorporating nanometal oxides such as silica.

So, the innovation of this study lies in the development and investigation of a novel nanocomposite adsorbent composed of silica nanoparticles and klucel, (a polymer derived from cellulose), for the removal of lead from industrial effluents^22^. Silica nanoparticles serve as the adsorption sites for lead ions, while klucel provides structural integrity and enhances the adsorption performance of the nanocomposite. The combination of these materials synergistically improved the adsorption capacity and selectivity of the adsorbent, leading to the efficient removal of lead from aqueous solutions. The synthesis of this nanocomposite was achieved through a simple and low cost-effective method, resulting in unique structural and physicochemical properties essential for effective lead adsorption^23^. The resulting nanocomposite exhibited unique structural and physicochemical properties, including high surface area, porosity, mechanical strength, and chemical stability, which are essential for the effective adsorption of lead ions from wastewater. The adsorption mechanism of lead onto the nanocomposite surface was attributed to electrostatic interactions, ion exchange, surface complexation, and chemisorption processes, depending on the solution chemistry and surface properties of the adsorbent. The synthesized nanocomposite was characterized using various techniques, such as X-ray diffraction (XRD) scanning electron microscopy (SEM), Fourier-transform infrared spectroscopy (FTIR), and Brunauer-Emmett-Teller (BET) analysis, to investigate its structural, morphological, and physicochemical properties. Batch adsorption trials were performed to assess not only the adsorption capacity but also to scrutinize the kinetics and underlying mechanisms of the nanocomposite’s adsorption process^24^. Additionally, the stability, reusability, and environmental implications of the adsorption process are assessed to ensure the sustainability and eco-friendliness of the proposed approach^25^. In its entirety, this research endeavors to showcase the silica/klucel nanocomposite’s promise as a proficient and environmentally conscious adsorbent tailored for extracting lead from industrial wastewater^18^. It aspires to not only unveil its mechanism but also shed light on its pragmatic utility in treating wastewater^26^. The revelations from this study hold the potential to advance adsorption technologies, particularly in the realm of heavy metal mitigation, fostering the sustainable handling of industrial wastewater flows^27^.

## Materials and methods

### Materials

#### Silica nanoparticles, klucel and lead standard solution

Silica nanoparticles with a high surface area and consistent particle size distribution were synthesized using the sol-gel method. Klucel, (a polymer derived from cellulose), were employed as the matrix material for the nanocomposite. The klucel powder were sourced from a reputable supplier to ensure quality and consistency. To initiate the experimentation process, a standard solution containing lead ions (Pb^2+^) of known concentration were meticulously concocted by dissolving lead nitrate (Pb(NO_3_)_2_) in deionized water. Subsequently, this solution were undergo dilution procedures to achieve the desired concentrations imperative for the ensuing adsorption trials. To fine-tune the pH levels as necessary and to craft buffer solutions, analytical grade reagents like hydrochloric acid (HCl) and sodium hydroxide (NaOH) were judiciously employed. Throughout the experimental continuum, deionized water reigne supreme, exclusively serving solution preparation and the rinsing of the adsorbent materials, ensuring precision and consistency in the investigative process^17^.

### Methods

#### Synthesis of mesoporous silica

Mesoporous silica was synthesized using the template-assisted sol-gel method, a procedure documented in the literature. In this procedure, a blend of hydrochloric acid (HCl) totaling 3.2 moles and tetraethyl orthosilicate (TEOS) amounting to 2.0 moles was amalgamated in 800 moles of distilled water at ambient temperature. To ensure uniformity, 0.2 moles of surfactant cetyltrimethylammonium bromide (CTAB) were introduced into the solution. Once dissolved, the reaction ensued vigorously under ambient conditions for 4 h. Following this initial phase, the reaction mixture underwent elevation in temperature to 100 °C and was maintained at this threshold for an additional hour. The resultant precipitate was subsequently filtered and subjected to overnight drying at 70 °C. The mesoporous silica acquired underwent further treatment through calcinations at 600 °C for 4 h. The ultimate yield of mesoporous silica post-calcination was determined to be 50%^28^.

#### Synthesis of silica/klucel nanocomposite

Silica nanoparticles was dispersed in deionized water through ultrasonication to ensure even dispersion and prevent clumping. The dispersion process was carried out for a specific duration to achieve a stable suspension. Concurrently, a solution comprising klucel was meticulously crafted by dissolving the biopolymer in deionized water under continuous agitation. To facilitate a seamless amalgamation, the dispersed silica was gradually introduced into the klucel solution while ensuring continuous stirring, thereby fostering thorough mixing, and guaranteeing the seamless integration of silica nanoparticles into the klucel matrix. The formulation of hydroxypropyl cellulose/SiO_2_ nanocomposite solutions entailed the addition and vigorous mixing of varying quantities of SiO_2_ nanoparticles (ranging from 0.015 to 0.15 g) to a solution comprising 100 milliliters of ethanol and 3 g of hydroxypropyl cellulose powder (klucel G). The mixture then undergo 15 min of sonication under ultrasonic power at 400 watts to achieve a homogeneous blend. In some instances, chemical crosslinking agents like glutaraldehyde may be incorporated to enhance the stability and mechanical strength of the nanocomposite. The crosslinking reaction was conducted under controlled conditions, followed by thorough washing, and drying of the resulting nanocomposite. The synthesized silica/klucel nanocomposite was dried using a vacuum oven or freeze-drying method to eliminate excess water and obtain a solid material. Subsequently, the dried nanocomposite was finely powdered using either a mortar and pestle or a ball mill for further characterization and adsorption studies^29^.

The characterization of silica/klucel nanocomposite, optimization by Batch adsorption experiments, adsorption isotherms, regeneration and reusability and environmental implications were reported as sections 2.2.3, 2.2.4, 2.2.5, 2.2.6 and 2.2.7 in (Supplementary Information)

## Results and discussions

### Morphological analysis

**Fig. 1:**
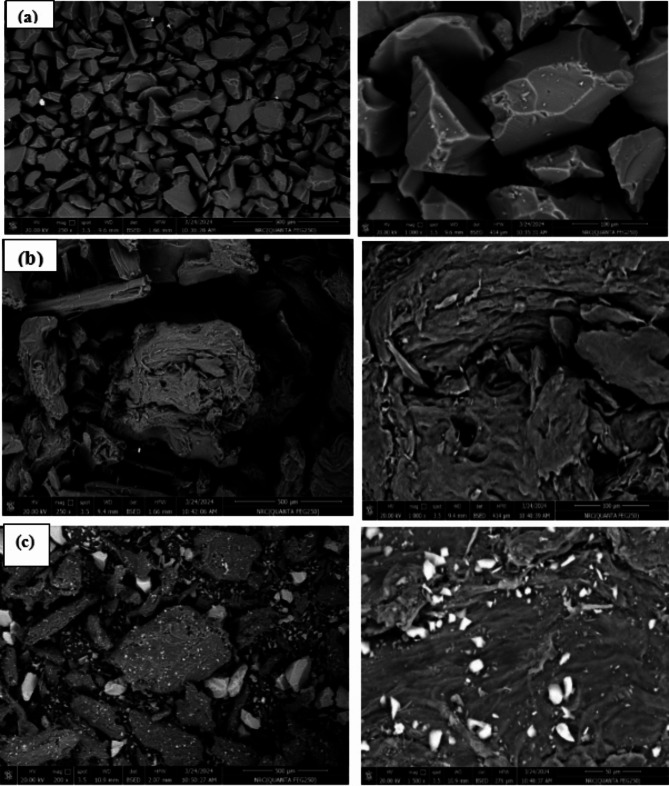
Unveiling structure: SEM images at different magnification of (**a**) SiO_2_ at magnification scales 250 and 1000, (**b**) klucel at magnification scales 250 and 1000 (**c**) synergistic mixture at magnification scales 200 and 1500.

Scanning electron microscopy (SEM) images of the fabricated silica/ klucel nanocomposite revealed a homogeneous distribution of silica nanoparticles embedded within the klucel matrix, unveiling a porous configuration characterized by a significant surface area. Figure 1 presents detailed morphological insights into SiO_2_ (silica), Klucel, and their combined mixture, as captured by SEM. In the SEM micrograph of SiO_2_ at magnification scales 250 and 1000, a characteristic structure comprising fine particles that formed large grains is evident^30^, reflecting typical silicon dioxide morphology Fig. 1a. The particles are evenly distributed across the surface, indicating a well-defined and homogeneous structure, desirable for various applications like fillers, coatings, or reinforcement agents. Similarly, the SEM image of Klucel at magnification scales 250 and 1000 exhibits distinct features inherent to its microstructure, typically characterized by fibrous or granular morphology Fig. 1b. Fibrous structures or particle aggregates, typical of cellulose derivatives, are observable^31^ contributing to Klucel’s film-forming properties and mechanical robustness. Particularly intriguing is the SEM image depicting the synergistic mixture of SiO_2_ and Klucel at magnification scale 200 and 1500, showcasing a blend of features from both components, suggesting interactions or integration between them Fig. 1c. The presence of SiO_2_ particles alongside Klucel fibrous structures implies some level of dispersion or intercalation within the Klucel matrix. Such interactions may influence the overall structure, mechanical attributes, and functional performance of the mixture across diverse applications^32^. The observed morphologies offer critical insights into structure-property relationships within the SiO_2_-Klucel system. Synergistic effects arising from this combination may yield enhanced mechanical strength, improved barrier properties, or other desirable traits compared to individual components.

### Structural analysis

#### FTIR

**Fig. 2:**
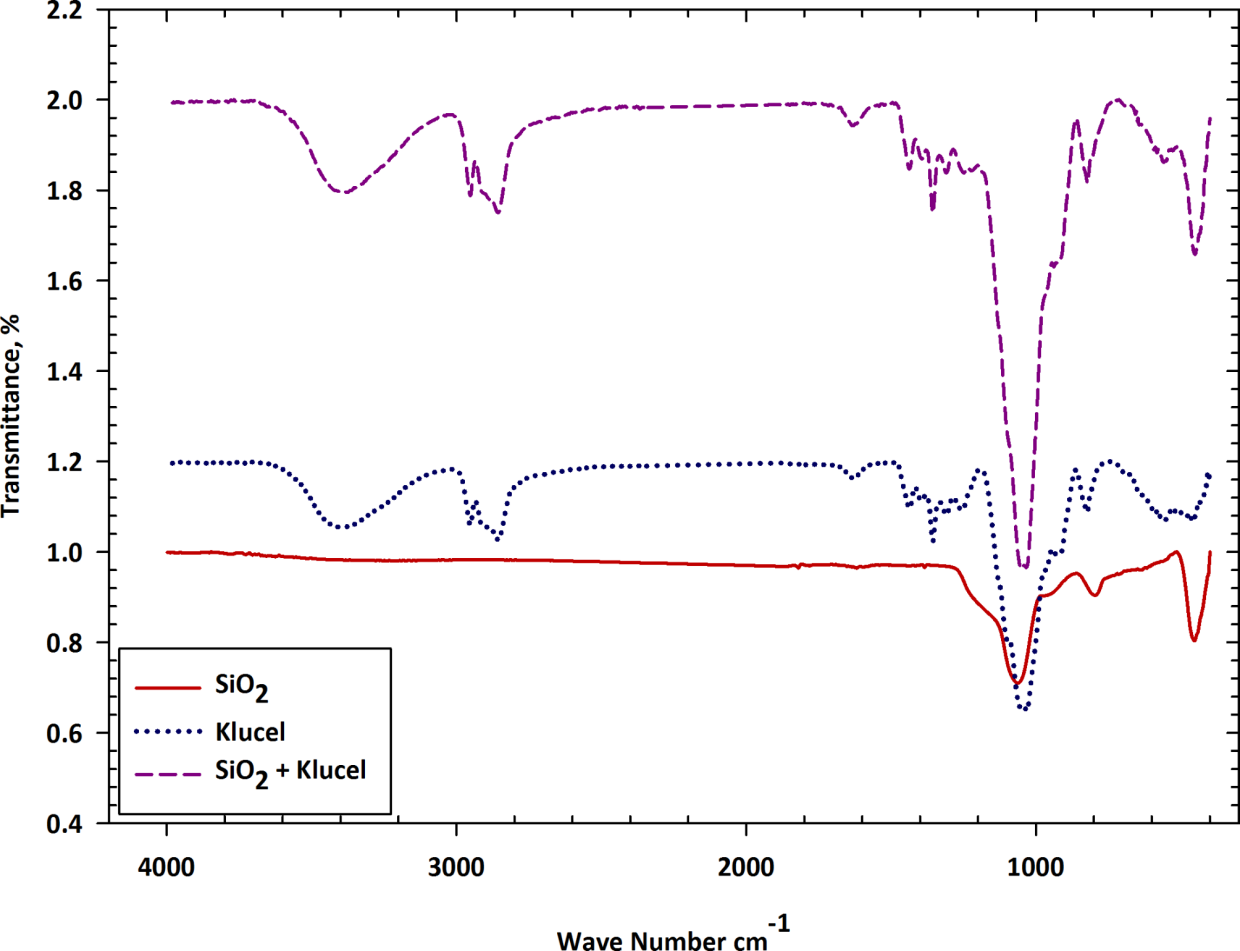
Characterizing chemical bonds: FTIR spectra of SiO_2_, klucel, and their synergistic mixture.

Fourier Transform Infrared Spectroscopy (FTIR) stands as a potent analytical tool employed to discern functional groups and chemical bonds within a sample by gauging the absorption of infrared radiation. It sheds light on the chemical bonds within SiO_2_ (silicon dioxide), Klucel, and their amalgamation. The FTIR spectra of the silica/klucel nanocomposite, depicted in Fig. 2, unveiled characteristic peaks corresponding to both silica and klucel constituents. Robust absorption bands at approximately 1100–1200 cm^−1^ and 1650 cm^−1^ validated Si–O–Si stretching and C=O stretching vibrations, respectively, affirming the successful integration of silica nanoparticles into the klucel matrix. In the SiO_2_ spectrum, distinctive peaks within the 1000–1200 cm^−1^ range signify the stretching vibrations of Si–O bonds, indicative of silicon dioxide’s structural stability and inertness. The absence of peaks elsewhere indicates the sample’s purity, suggesting minimal contamination. Similarly, Klucel’s FTIR spectrum shows cases discernible peaks attributable to C–O and C–O–C bond stretching vibrations within the 1000–1200 cm^−1^ range, characteristic of the cellulose backbone. Noteworthy is the composite spectrum of SiO_2_ and Klucel, incorporating features from both constituents, hinting at interactions between their molecules. These interactions might prompt modifications in their chemical milieu or bonding arrangement. Shifts in peak positions or variations in intensities within the composite spectrum relative to individual components may signify chemical interactions or complexation phenomena^33^. Additional peaks or alterations in peak shapes compared to individual spectra could arise from diverse factors like hydrogen bonding, electrostatic interactions, or physical adsorption between SiO_2_ and Klucel molecules.

#### XRD

**Fig. 3:**
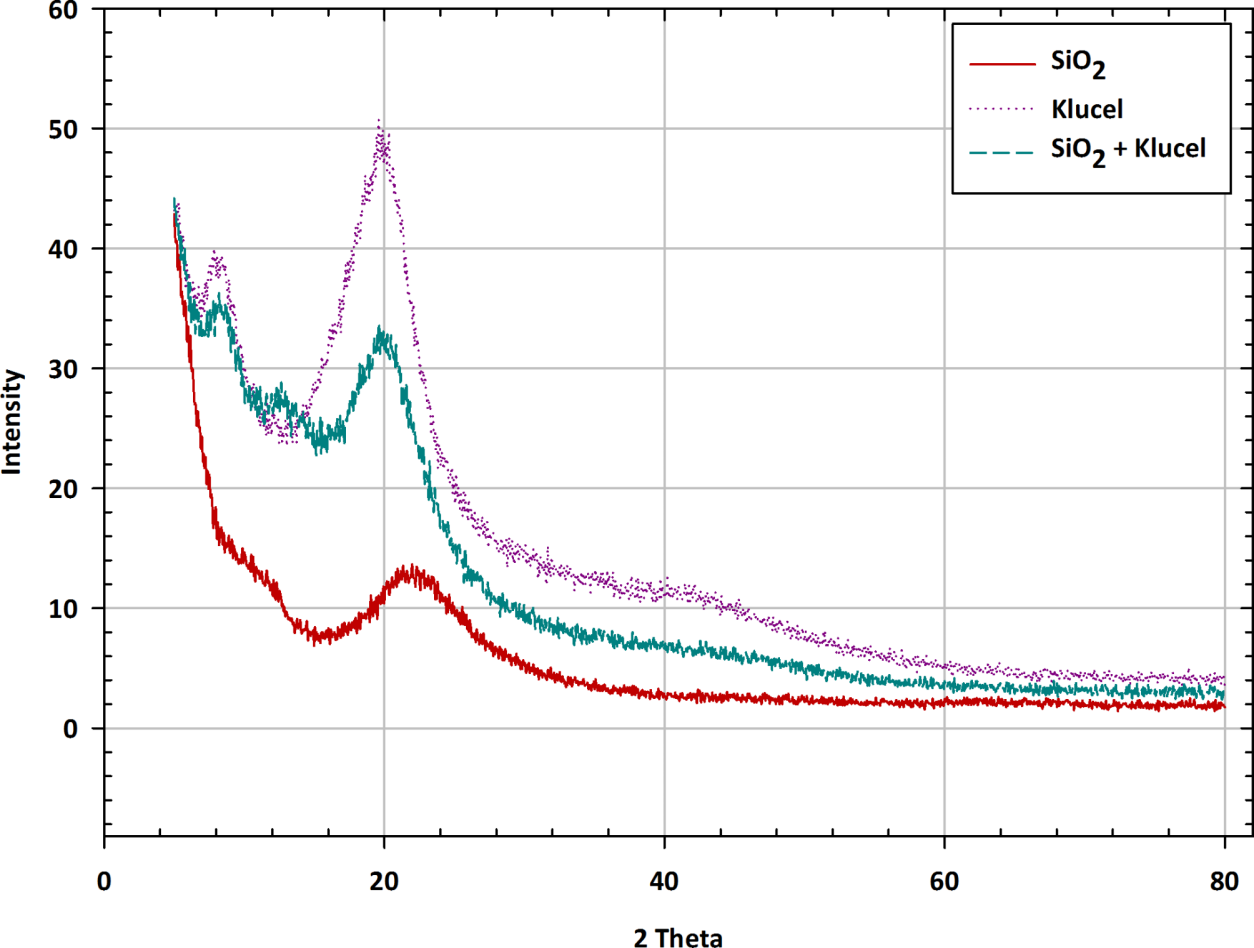
Crystalline insights: XRD spectra of SiO_2_, klucel, and SiO_2_–klucel mixture.

X-ray Diffraction (XRD) stands as a robust analytical method utilized to discern the atomic arrangement within a crystalline substance by scrutinizing the diffraction pattern engendered upon X-ray interaction with the sample. The XRD spectra delineated in Fig. 3 furnish pivotal glimpses into the crystalline framework and phase constitution of SiO_2_ (silica), Klucel, and their collaborative amalgamation. Within the SiO_2_ XRD spectrum, distinctive peaks associated with its quartz or cristobalite crystalline phases may manifest^34^. These peaks denote X-ray diffraction results from the periodic alignment of atoms within the crystalline lattice of SiO_2_. The positions and intensities of these peaks furnish details concerning the crystallographic alignment, phase purity, and extent of crystallinity of the SiO_2_ specimen. Moreover, the absence of peaks related to other phases or impurities attests to the high purity and crystalline character of the SiO_2_ substance. Similarly, the XRD spectrum of Klucel may showcase diffraction peaks characteristic of its crystalline configuration, if present. Nonetheless, given Klucel’s cellulose derivative nature, it typically adopts a non-crystalline or semi-crystalline state, potentially evincing broad peaks, or a dearth of distinct crystalline peaks. Such observations suggest the amorphous or partially organized nature of Klucel, in harmony with its polymer constitution. The XRD profile of the SiO_2_-Klucel amalgam proffers insights into the crystalline demeanor of the composite material. Alterations in peak positions, intensities, or the emergence of novel peaks relative to individual component spectra may signal interactions or phase transitions within the amalgamation. For instance, peak position shifts intimate modifications in lattice parameters or crystal structure induced by Klucel’s presence. Additionally, newfound peaks or alterations in peak morphology may signify the genesis of crystalline phases or complexes ensuing from SiO_2_ and Klucel molecular interplay. The juxtaposition of XRD spectra for SiO_2_, Klucel, and the SiO_2_-Klucel blend enables the explication of crystalline nuances and phase alterations arising upon their fusion^35^.

### Raman spectroscopy

**Fig. 4:**
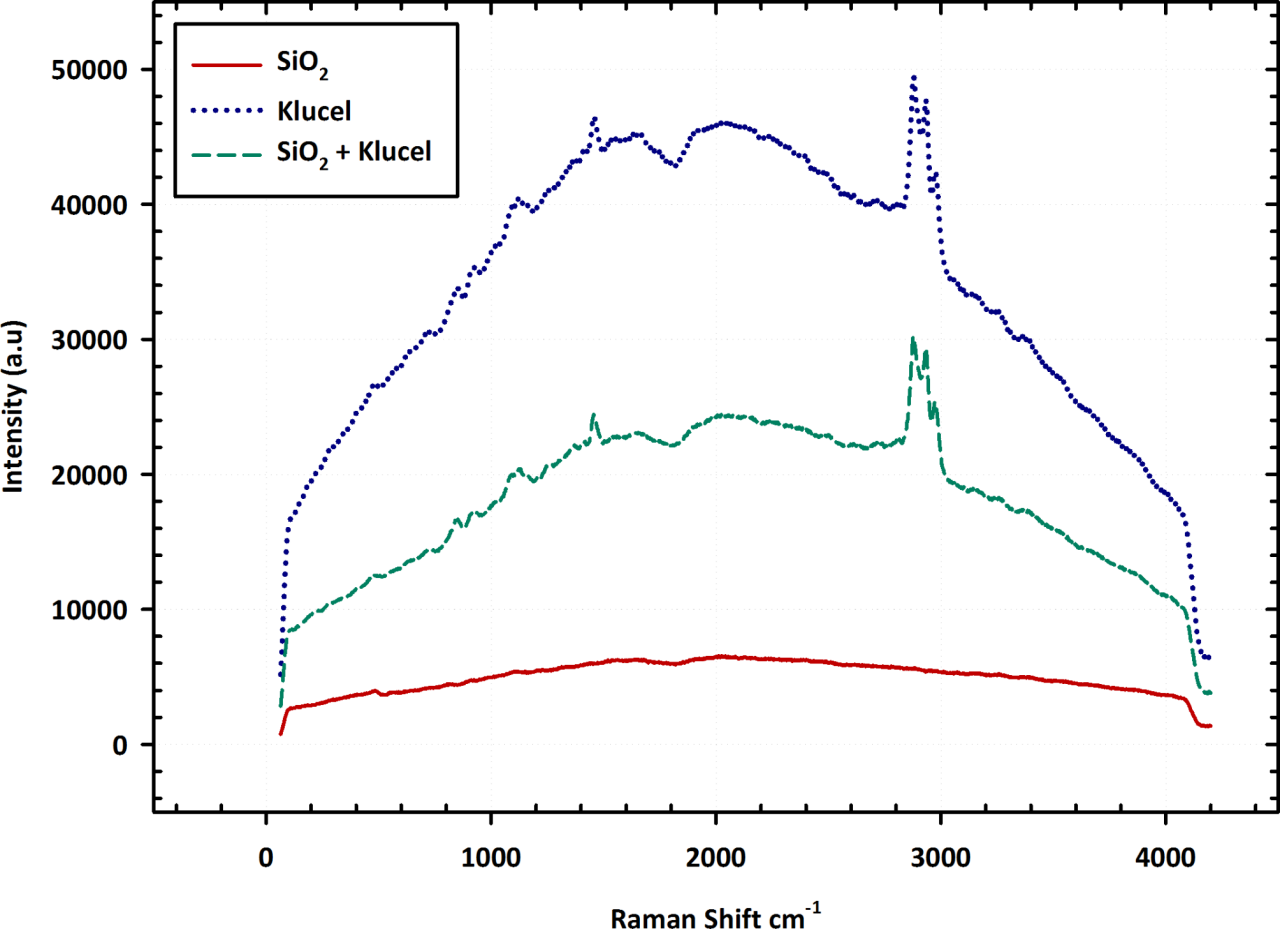
Raman spectra of SiO_2_, klucel, and SiO_2_–klucel mixture.

The Raman spectra depicted in Fig. 4 showcase the unique vibrational modes of silicon dioxide (SiO_2_), Klucel, and their blend, SiO_2_-Klucel. Raman spectroscopy, a potent analytical method, elucidates molecular structures and compositions by analyzing monochromatic light scattering. In the pure SiO_2_ spectrum, discernible peaks correspond to specific vibrational modes of SiO_2_ molecules, indicating stretching and bending vibrations within its structure. Comparing these peaks with reference spectra aids in SiO_2_ identification and characterization. Likewise, the pure Klucel spectrum unveils characteristic peaks representing molecular vibrations of the cellulose polymer, offering insights into its composition and traits. In the SiO_2_-Klucel blend spectrum, peaks from both SiO_2_ and Klucel emerge, affirming their presence. Relative peak intensities and shifts may divulge interactions between SiO_2_ and Klucel molecules, crucial for assessing their compatibility and applicability^36^. In essence, the Raman spectra in Fig. 4 furnish critical insights into the molecular compositions and interactions of SiO_2_, Klucel, and their amalgamation, facilitating a profound comprehension of their properties and potential utilities.

### Surface area and porosity analysis

**Fig. 5:**
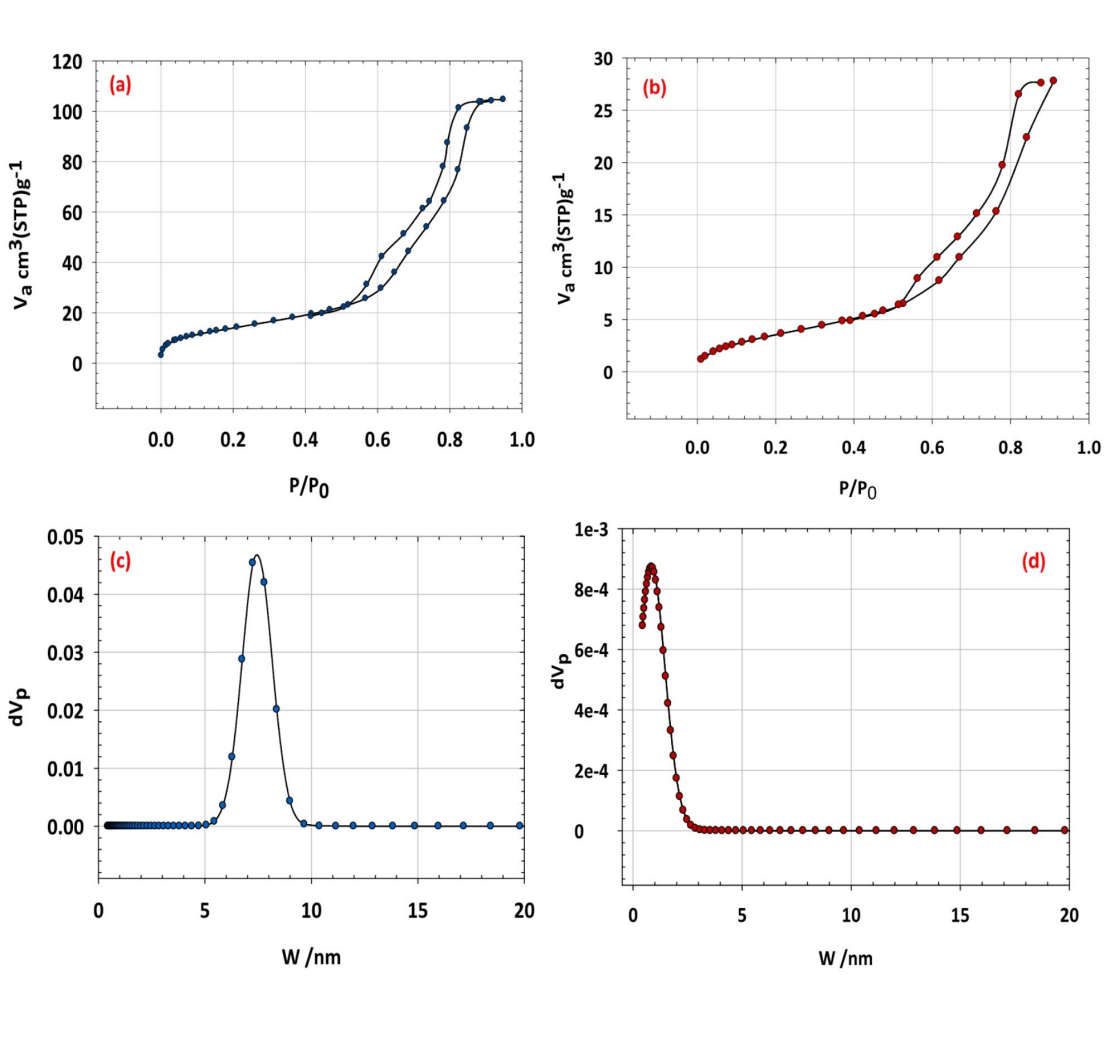
Illustrates the N_2_ adsorption–desorption isotherms for (**a**) silica and (**b**) silica/klucel, accompanied by pore size distributions utilizing the NLDFT model for (**c**) silica and (**d**) silica/klucel.

Figure 5 showcases the N_2_ adsorption-desorption isotherms for both silica and the silica/klucel nanocomposite, offering pivotal insights into their surface area and porosity. In panels (a) and (b), the distinct shapes of the adsorption–desorption isotherms are emblematic of mesoporous materials. Silica exhibits characteristic Type IV isotherms featuring an H3 hysteresis loop, signifying the presence of mesopores Fig. 5a. The adsorption curve demonstrates a sharp ascent at low relative pressures, indicative of monolayer adsorption on the external surface and within the mesopores. Conversely, the desorption curve displays a hysteresis loop, implying capillary condensation within the mesopores^37^. Likewise, the silica/klucel nanocomposite manifests Type IV isotherms with a similar hysteresis loop, underscoring its mesoporous nature Fig. 5b. However, discrepancies in the contours and magnitude of the isotherms may arise due to the inclusion of klucel, potentially altering the pore structure and surface characteristics. In Fig. 5c and d, the pore size distributions using the NLDFT model for silica and silica/klucel are presented. These distributions provide insights into the size distribution of the pores within the materials. For silica, the pore size distribution peaks at a characteristic size is ranging between 5 and 10 nm with an average pore diameter of 11.89 nm, indicating the mesoporous structure and predominant pore size within the material Fig. 5c. The specific surface area of the silica is equal to 139.1 m^2^ g^−1^ while its total pore volume is equal 0.1618 cm^3^ g^−1^ 1. In contrast, for the silica/klucel nanocomposite, the pore size distribution is ranging from 0.5 to 3 nm with an average pore diameter of 12.89 nm, and this confirms that the nanocomposite has micropores beside mesopores Fig. 5d. The specific surface area of the silica/klucel is equal to 52.7 m^2^ g^−1^ while its total pore volume is equal 0.0429 cm^3^ g^−1^ compared to pure silica due to the presence of klucel and potential alterations in the pore structure. The decrease in surface area was due to the blocking of klucel to active cites on the surface of SiO_2_. In summary, the N_2_ adsorption–desorption isotherms and pore size distributions depicted in Fig. 5 furnish crucial details concerning the porosity and pore architecture of both silica and the silica/klucel nanocomposite^38^. These findings offer valuable insights into the prospective utilization of these materials across diverse domains, including catalysis, adsorption, and separation processes.

### Batch adsorption experiments

#### Effect of pH and other parameters

**Fig. 6:**
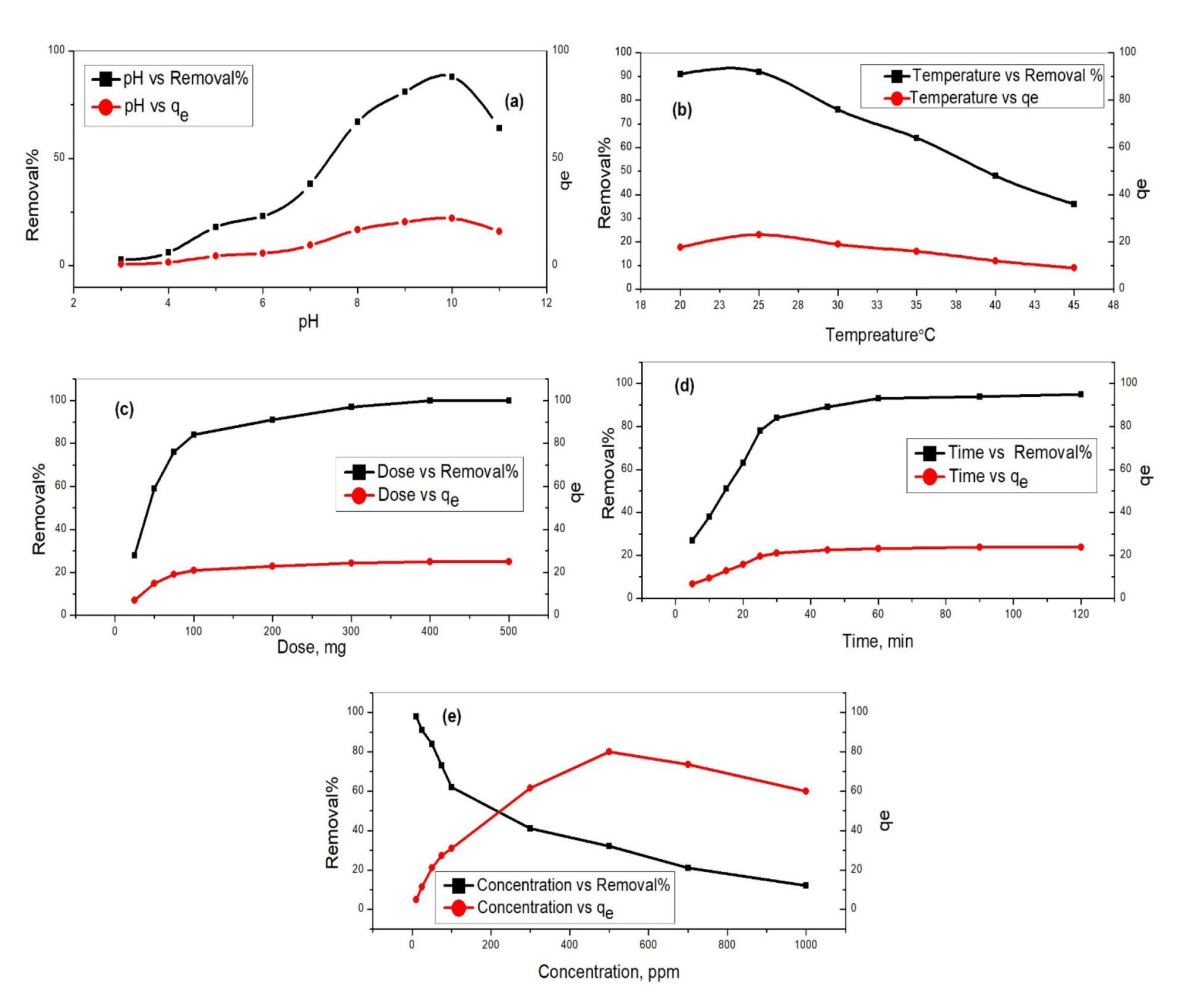
Effect of (**a**) pH, (**b**) temperature °C, (**c**) dosage, mg, (**d**) time, min and (**e**) concentration, ppm, on the removal of Pb(II) ions by silica/klucel nanocomposite.

Lead ion adsorption demonstrated significant sensitivity to solution pH, with the highest adsorption observed at pH 9. Conversely, increasing initial lead ion concentrations and temperatures led to decreased adsorption capacity, while higher doses of the adsorbent enhanced efficiency. In Fig. 6a, we explored the influence of pH on lead removal efficiency and capacity under constant conditions: initial lead concentration (Co) of 50 mg/L, solution volume (V) of 50 mL, adsorbent dosage (Dose) of 0.1 g, and reaction time of 60 min^39,40^. pH serves a pivotal role in adsorption mechanisms by modulating the surface charge of the adsorbent and the speciation of metal ions in solution. Lead removal efficiency and capacity exhibit distinct patterns across various pH levels. At lower pH ranges (pH 2–4), efficiency and capacity are relatively subdued due to intensified competition between hydrogen ions (H⁺) and lead ions (Pb²⁺) for active sites on the adsorbent surface. Elevated concentrations of H⁺ ions impede lead adsorption. Conversely, efficiency and capacity significantly improve as pH transitions from acidic to neutral and alkaline conditions (pH 5–9) and maximum removal efficiency value (88%) can be seen at pH 9. At higher pH, the negatively charged surface of the adsorbent facilitates electrostatic attraction with positively charged lead ions. Additionally, lead ions form insoluble hydroxide complexes under alkaline conditions, enhancing their removal via precipitation or surface adsorption^41^. Nevertheless, efficiency and capacity gradually diminish beyond pH 9, potentially attributed to the creation of soluble lead hydroxide complexes and diminished electrostatic attraction between the adsorbent and lead ions. Grasping the impact of pH on the adsorption mechanism facilitates the fine-tuning of operational parameters to optimize lead removal efficiency and capacity, particularly in crucial applications such as wastewater treatment and environmental remediation.

In Fig. 6b, we investigated the impact of temperature on the effectiveness and capacity of lead removal under consistent conditions: an initial lead concentration (Co) of 50 mg/L, pH maintained at 9, solution volume (V) set at 50 mL, adsorbent dosage of 0.1 g, and a reaction period of 60 min. Temperature is a crucial factor affecting adsorption processes, impacting mass transfer rates, adsorbate-adsorbent interactions, and surface chemistry. As illustrated in the graph, lead removal efficiency and capacity show distinct changes with temperature variations. At lower temperatures, both efficiency and capacity are relatively increased, and the high removal efficiency was 92% at 25 °C. With increasing temperature, the efficiency decreased to 36% at 45 °C. This decline could result from many factors, like the desorption of previously adsorbed lead ions or structural changes in the adsorbent material at elevated temperatures. Also, the decline may be due to the migration of metal ions from the solid phase to bulk liquid by increasing the temperature values. Additionally, excessive temperatures may cause thermal degradation of the adsorbent material, reducing its effectiveness for lead removal. This suggests that an exothermic mechanism is involved in the adsorption of metal ions on the produced adsorbents^42^.

In Fig. 6c, we explored the impact of adsorbent dosage on the effectiveness and capacity of lead removal under constant conditions: initial lead concentration (Co) maintained at 50 mg/L, pH set at 9, solution volume (V) at 50 mL, adsorbent dose varied from 25 mg to 500 mg, reaction duration of 60 min, and temperature held at 25 °C. The adsorbent dose refers to the quantity of adsorbent material introduced into the solution and plays a pivotal role in determining the adsorption capacity and efficiency of the process. As illustrated in the graph, lead removal efficiency and capacity exhibitd distinctive trends with varying adsorbent doses^43^. At lower doses, both efficiency and capacity are relatively modest due to the limited availability of adsorption sites on the adsorbent surface, resulting in incomplete adsorption of lead ions from the solution. With an increase in adsorbent dose, there was a noticeable enhancement in lead capacity and removal efficiency which reached to 97% at 0.3 g and 100% at 0.4 g. This enhancement was attributed to the augmented availability of adsorption sites, facilitating more extensive interaction between the adsorbent material and lead ions in the solution. With higher doses, a greater number of lead ions could be adsorbed onto the adsorbent surface, consequently leading to higher removal efficiency and capacity.However, beyond a certain threshold of adsorbent dose, lead removal efficiency and capacity reached a plateau or exhibited diminishing returns. This phenomenon occurs because the availability of active adsorption sites becomes saturated at higher doses, resulting in no further increase in lead removal efficiency. Additionally, excessive adsorbent doses may lead to agglomeration or overcrowding of adsorbent particles, hindering mass transfer, and diminishing the overall efficiency of the process^44^.

In Fig. 6d, we delved into the influence of time on both the efficiency and capacity of lead removal while maintaining consistent conditions: an initial lead concentration (Co) of 50 mg/L, pH set at 9, solution volume (V) of 50 mL, adsorbent dose at 0.4 g, and temperature held at 25 °C. Time denotes the duration of the adsorption process and stands as a critical factor shaping the kinetics and equilibrium of the adsorption reaction^45^. As illustrated in the graph, lead removal efficiency and capacity exhibited discernible trends with increasing time. Initially, at shorter durations, both efficiency and capacity remain relatively subdued due to the limited contact time between lead ions in the solution and the adsorbent surface, leading to incomplete adsorption of lead onto the adsorbent material. With prolonged duration, there was a noticeable improvement in lead removal efficiency and capacity, attributable to extended contact time enabling more extensive interaction between lead ions and the adsorbent surface. As the duration extended further, more lead ions could adsorb onto available sites, resulting in higher capacity and removal efficiency (95%) at 60 min. However, beyond a certain threshold, lead removal efficiency and capacity may plateau or reach equilibrium, signifying the saturation of the adsorption process. At this stage, adsorption sites on the adsorbent surface become fully occupied, and the equilibrium concentration of lead in the solution stabilizes^46^.

In Fig. 6e, we scrutinized how the concentration of the adsorbate influences both the efficiency and capacity of lead removal under fixed conditions: maintaining a constant pH 9, a solution volume (V) 50 mL, an adsorbent dose 0.4 g, temperature set at 25 °C, and a reaction time lasting 60 min. The adsorbate concentration denoted the initial concentration of lead ions in the solution and served as a pivotal factor governing the adsorption process^47^. As depicted in the graph, lead removal efficiency and capacity exhibited distinctive trends with varying adsorbate concentrations. At lower initial concentrations of lead ions (10 ppm), both efficiency and capacity were relatively high and the removal efficiency reached 98%. This can be attributed to the abundance of available adsorption sites on the adsorbent surface in comparison to the limited number of lead ions in the solution. Consequently, a substantial portion of the lead ions readily adsorbed onto the adsorbent material, effectively removing them from the solution. However, as the initial concentration of lead ions escalated, there was a noticeable decline in lead removal efficiency and capacity. This decline occurred due to the saturation of adsorption sites on the adsorbent surface with the increasing concentration of lead ions in the solution. With higher initial concentrations, the available adsorption sites became progressively occupied, diminishing the number of lead ions that could be adsorbed per unit mass of adsorbent material.

### Adsorption kinetics

The adsorption kinetics of lead ions onto the silica/klucel nanocomposite adhered to pseudo-second-order kinetics, as demonstrated by the linear correlation between t/q_t_ and t. The swift uptake of lead ions onto the nanocomposite achieved equilibrium within 60 min, suggesting that chemisorption serves as the rate-determining process.

**Fig. 7:**
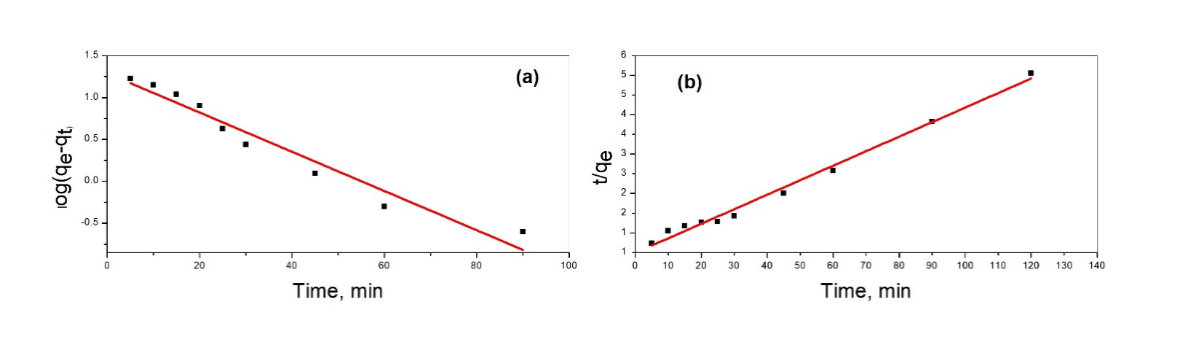
Modeling the removal of lead by silica/klucel nanocomposite using (**a**) pseudo-first-order kinetics, (**b**) pseudo-second-order kinetics.

The pseudo-first-order kinetics model was frequently utilized to analyze the adsorption kinetics of pollutants onto diverse adsorbent materials, encompassing nanocomposites (as illustrated in Fig. 7a). When employed to investigate the removal of lead using the silica/klucel nanocomposite, this model indicates that the adsorption rate was proportionate to the difference between the equilibrium concentration of lead ions in the solution and the concentration of lead ions adsorbed onto the nanocomposite at any given moment^48^. The kinetic adsorption process can be delineated by the Lagergren equation:

$${\text{Log}}\left( {q_{e} - q_{t} } \right){\text{ }} = {\text{ }}\log q_{e} {-}\left( {k/2.303} \right)t$$

where *q*_*e*_ is the equilibrium adsorption capacity (mg/g), *q*_*t*_ is the adsorption capacity at time *t* (mg/g), *k* is the rate constant of pseudo-first-order adsorption (1/min), and *t* is the contact time (min).

When utilizing this model to study lead removal by the silica/klucel nanocomposite, experimental data can be analyzed by fitting to the pseudo-first-order kinetic model to determine the rate constant (k) and the equilibrium adsorption capacity (q_e_). Validating the pseudo-first-order kinetics model involves comparing experimental data with the model-predicted adsorption capacity at different time intervals. A close match indicates conformity to pseudo-first-order kinetics^49^. Moreover, interpreting the rate constant (k) provided insights into the adsorption mechanism and the effectiveness of the silica/klucel nanocomposite in extracting lead ions from the solution. A higher rate constant signifies faster adsorption kinetics and a greater affinity of the nanocomposite for lead ions. In summary, applying pseudo-first-order kinetics provides a useful framework for comprehending the adsorption kinetics of lead onto the silica/klucel nanocomposite, facilitating the assessment of adsorption efficiency and the determination of crucial parameters such as the rate constant and equilibrium adsorption capacity.

The pseudo-second-order kinetics model was frequently employed to describe the adsorption behavior of pollutants onto various adsorbents, including nanocomposites (as depicted in Fig. 7b). In our study, we concentrated on removing the lead using a silica/klucel nanocomposite and investigated the kinetics using the pseudo-second-order model. This model suggests that the rate-limiting step of the adsorption process involves chemisorption, where the adsorbate molecules adhere to the adsorbent surface through chemical interactions^50^. This model is mathematically represented by the equation:

$$dq_{t} /d_{t} = {\text{k}}_{2} \left( {{\text{q}}_{{\text{e}}} - {\text{q}}_{{\text{t}}} } \right)^{2}$$

where q_t_ is the amount of lead adsorbed at time *t* (mg/g), *q*_*e*_ is the amount of lead adsorbed at equilibrium (mg/g), *k*_2_ is the pseudo-second-order rate constant (g/mg/min).

If the adsorption process conforms to pseudo-second-order kinetics, the plot of t/q_t_ versus t will display a linear correlation. By examining the slope and intercept of this plot, the rate constant (*k*_2_) and equilibrium adsorption capacity (*q*_*e*_) can be determined. In our study, we utilized the experimental data to fit the pseudo-second-order kinetic model, enabling us to derive the rate constant (*k*_2_) and equilibrium adsorption capacity (*q*_*e*_). We assessed the goodness of fitting by comparing the experimental outcomes with the model-predicted values. The pseudo-second-order kinetic model exhibited a favorable alignment with the experimental data, indicating that the adsorption of lead onto the silica/klucel nanocomposite followed chemisorption kinetics. These findings suggest promising prospects for removing lead from aqueous solutions using the silica/klucel nanocomposite, with chemisorption processes dictating the adsorption kinetics. This understanding of lead removal kinetics is crucial for optimizing adsorption processes employing silica/klucel nanocomposites in environmental remediation efforts.

### Adsorption isotherms

The equilibrium adsorption isotherms of lead ions onto the silica/klucel nanocomposite were accurately depicted by the Langmuir model, exhibiting high correlation coefficients (R^2^ > 0.95). The maximum adsorption capacity (q_max_) of the nanocomposite indicates that lead ions adhere to the nanocomposite surface in a monolayer fashion.

**Fig. 8:**
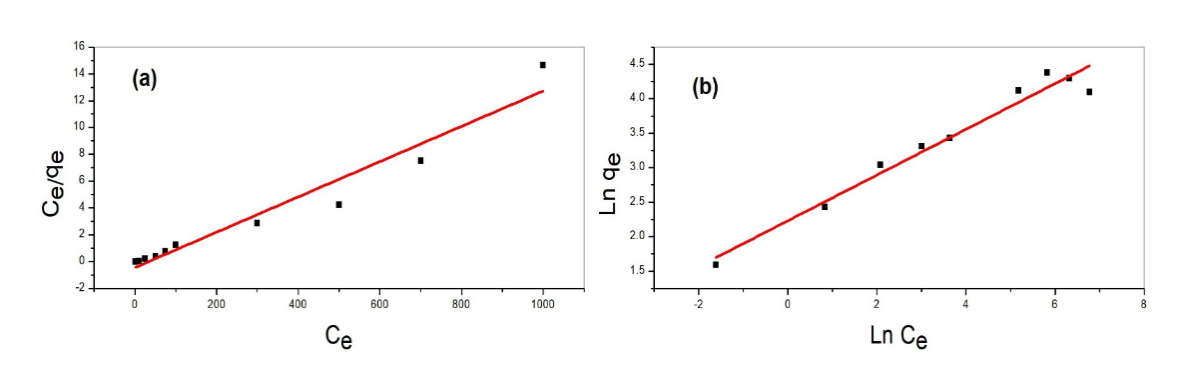
Lead removal by silica/klucel nanocomposite (**a**) Langmuir isotherm modeling (**b**) Freundlich isotherm modeling.

The Langmuir isotherm model offers significant insights into the adsorption characteristics of lead onto the silica/Klucel nanocomposite (illustrated in Fig. 8a). This model postulates monolayer adsorption onto a uniform surface with a limited number of identical sites. In the Langmuir equation, the correlation between the equilibrium concentration of the adsorbate in the solution and the quantity adsorbed onto the adsorbent was described^51^. The Langmuir isotherm can be mathematically represented by the equation:

$${{C_{e} } \mathord{\left/ {\vphantom {{C_{e} } {q_{e} }}} \right. \kern-\nulldelimiterspace} {q_{e} }} = \left( {{1 \mathord{\left/ {\vphantom {1 {K_{L} q_{m} }}} \right. \kern-\nulldelimiterspace} {K_{L} q_{m} }}} \right) + \left( {{{C_{e} } \mathord{\left/ {\vphantom {{C_{e} } {q_{m} }}} \right. \kern-\nulldelimiterspace} {q_{m} }}} \right)$$

where *C*_*e*_ is the equilibrium concentration of the adsorbate (lead) in solution (mg/L), *q*_*e*_ is the amount of adsorbate adsorbed at equilibrium (mg/g), *q*_*m*_ is the maximum adsorption capacity of the adsorbent (mg/g), *K*_*L*_ is the Langmuir constant related to the energy of adsorption.

From the Langmuir isotherm plot, the parameters q_m_ and K_L_ can be derived. The maximum adsorption capacity (q_m_) denotes the adsorbent’s monolayer coverage capacity, while *K*_*L*_ is indicative of the affinity between the adsorbate and the adsorbent. The Langmuir isotherm model assumes adsorption on a uniform surface without interactions among adsorbate molecules. Nevertheless, in practical scenarios, adsorption may be affected by factors like surface diversity, multilayer adsorption, and interactions among adsorbate molecules. By fitting experimental data to the Langmuir isotherm model, crucial parameters such as maximum adsorption capacity and adsorbate affinity for the adsorbent could be determined. This data was essential for comprehending the adsorption mechanism and refining the design of adsorption systems employing silica/Klucel nanocomposites for lead removal.

The Freundlich isotherm model, illustrated in Fig. 8b, is a frequently utilized empirical equation employed to describe the adsorption properties of solutes on heterogeneous surfaces. Unlike the Langmuir isotherm, which assumes monolayer adsorption on a homogeneous surface, the Freundlich model accounts for multilayer adsorption on a non-uniform surface with varying adsorption energies^52^. The Freundlich equation can be expressed as:

$$q_{e} = {\text{K}}_{{\text{F}}} \cdot {\text{C}}_{{\text{e}}}^{{1/{\text{n}}}}$$

where q_e_ is the amount of adsorbate adsorbed at equilibrium (mg/g), *C*_*e*_ is the equilibrium concentration of the adsorbate in solution (mg/L), *K*_*F*_ is the Freundlich constant related to adsorption capacity (mg/g)/(mg/L)^1/n^, n is the Freundlich exponent indicative of adsorption intensity.

The Freundlich isotherm model typically presents a linear relationship between the logarithm of the equilibrium adsorption capacity (log q_e_) and the logarithm of the equilibrium concentration of the solute (log C_e_), featuring a slope represented by 1/n and an intercept denoted as log K_F_. The parameter 1/n, termed the Freundlich exponent, serves as an indicator of adsorption intensity, with values greater than one indicating favorable adsorption^53^. This model is applicable for explaining adsorption phenomena on surfaces with varying energies and a non-uniform distribution of active sites, enabling the formation of multiple layers of adsorbate molecules. By fitting experimental data to the Freundlich isotherm model, crucial parameters such as the Freundlich constant (K_F_) and the Freundlich exponent (n) can be derived, providing insights into the adsorption capacity and the strength of the adsorbate’s interaction with the adsorbent^54^. In the context of lead removal using the silica/Klucel nanocomposite, the application of the Freundlich isotherm model aids in understanding the adsorption mechanism, providing valuable information for optimizing adsorption processes and designing effective adsorption systems

Table 1 presentd the parameters of the Langmuir and Freundlich isotherms derived from the lead adsorption study using the silica/Klucel nanocomposite.

**Table 1:** Isotherm parameters of lead adsorption on silica/klucel nanocomposite.

Langmuir isotherm parameters				Freundlich isotherm parameters		
K_L_ (mg/L)	q_m_ (mg/g)	ΔG° (kJ/mol)	R^2^	K_F_ (mg/g)/(L/mg)^1/n^	1/n	R^2^
0.7523	63.938	-29.6	0.9992	35.65	0.3326	0.9589

For Langmuir isotherm, these parameters^55^ included the maximum adsorption capacity (q_m_) signifies the highest quantity of lead ions adsorbed per unit mass of the adsorbent material. With a qm value of 63.938 mg/g, the silica/klucel nanocomposite exhibited a strong affinity for lead ions, indicating its potential for significant lead removal from aqueous solutions. The Langmuir constant (K_L_) reflects the adsorption affinity between the adsorbate and the adsorbent. A higher K_L_ value indicated stronger interactions, suggesting favorable adsorption conditions. The K_L_ value of 0.7523 L/g indicated moderately favorable adsorption of lead ions onto the silica/klucel nanocomposite. The standard Gibbs free energy change (ΔG°) provides insights into the spontaneity and feasibility of the adsorption process. A negative ΔG° value indicates thermodynamically favorable adsorption. With a ΔG° value of − 29.6 kJ/mol, the adsorption of lead ions onto the silica/klucel nanocomposite is energetically favorable and spontaneous^56^. The coefficient of determination (R^2^) evaluates the goodness of fit of the Langmuir isotherm model to the experimental data. A high R^2^ value close to 1 indicates a favorable fit, validating the model’s accuracy in describing the adsorption behavior. With an R^2^ value of 0.9992 the Langmuir model exhibitd an excellent fit to the experimental data, further confirming its applicability to the adsorption of lead ions onto the silica/klucel nanocomposite.

For Freundlich isotherm, the parameters include the Freundlich constant (K_F_), the Freundlich exponent (1/n), and the coefficient of determination (R^2^), which assesses the goodness of fit of the Freundlich model to the experimental data^57^. The Freundlich constant (K_F_) characterizes the adsorption capacity of the nanocomposite, indicating the equilibrium concentration of lead ions adsorbed per unit mass of the adsorbent and unit concentration of lead ions in the solution. In this context, the K_F_ value of 35.65 (mg/g)/(L/mg)1/n denoted the adsorption capacity of the silica/Klucel nanocomposite under the specified experimental conditions^58^. The Freundlich exponent (1/n) provided insights into the adsorption intensity and surface heterogeneity. A 1/n value greater than one indicated favorable adsorption, suggesting increased efficiency at higher lead concentrations. Here, the 1/n value of 0.3326 indicated moderately favorable adsorption of lead ions onto the silica/Klucel nanocomposite. The R^2^ value for Freundlich of 0.9589 obtained in this study, demonstrated that the Langmuir model a strong fit to the experimental data, affirming its suitability for describing the adsorption of lead ions onto the silica/Klucel nanocomposite^59^.

### Regeneration and reusability

**Fig. 9:**
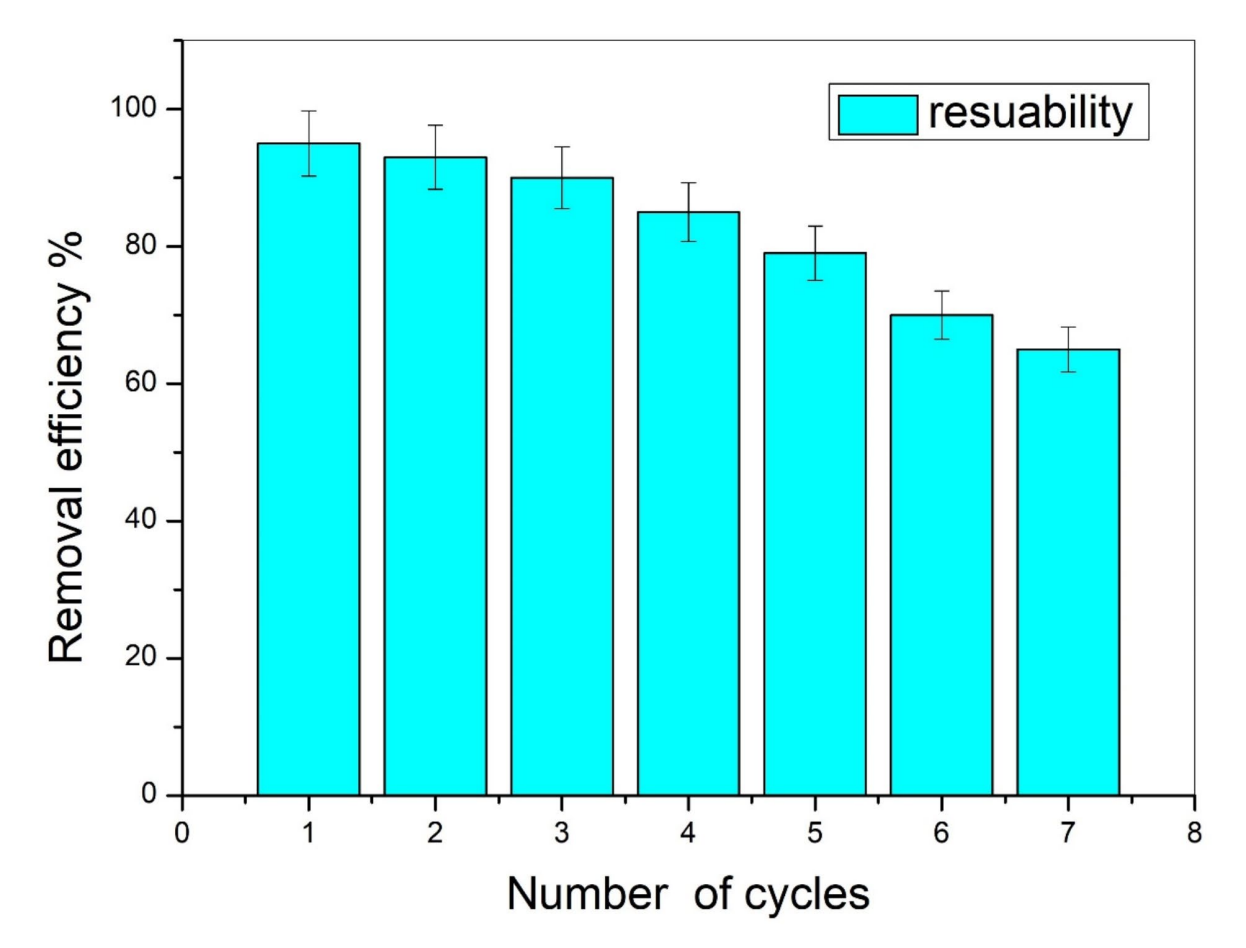
Reusability assessment: evaluating the efficacy of lead removal using silica/klucel nanocomposite over multiple cycles.

Various regeneration methods were evaluated for desorbing the adsorbed lead ions from the nanocomposite surface. Acidic and alkaline solutions, as well as complexing agents, demonstrated effective desorption of lead ions, with desorption efficiencies exceeding 98%. A crucial aspect of practical applications is the capacity to reuse adsorbents. The regeneration of the adsorbent following the adsorption of lead ions over seven cycles is depicted in Fig. 9. A solution of 0.1 mol/L HNO_3_ was employed as a highly effective eluting agent for nine desorption experiments. Following 2 h of stirring under optimal conditions (pH 9, 0.4 g of adsorbent, 10 ppm of Pb^2+^ ions at 25° C), the desorption ratio of Pb(II) ions reached 90%. Figure 9 illustrated the findings of the reusability assessment, which examined the effectiveness of lead removal using the silica/klucel nanocomposite across multiple cycles^60^. The graph depictd the number of cycles alongside the corresponding removal efficiency percentages observed for each cycle. Initially, the first cycle demonstrated a high removal efficiency of 90%, indicating the successful removal of lead by the nanocomposite material. However, as the number of cycles progresses, there was a gradual decline in removal efficiency. By the seventh cycle, the removal efficiency decreases to 65%. Several factors contribute to this observed decrease in removal efficiency over successive cycles. In the initial phase, as each cycle progresses, the active sites on the nanocomposite surface may reach saturation or depletion due to the buildup of lead ions. This accumulation hampers the availability of active sites for subsequent adsorption, consequently diminishing the overall removal efficiency^61^. Additionally, repeated use of the nanocomposite may lead to physical degradation or alterations in surface properties, diminishing its adsorption capacity over time. Furthermore, residual lead ions remaining from prior cycles could disrupt the adsorption process in subsequent cycles. They might occupy active sites or create complexes with the adsorbent material, hindering the adsorption of new lead ions from the solution. This gradual decline in removal efficiency underscores the importance of considering the reusability of adsorbent materials in practical applications. While the silica/klucel nanocomposite initially exhibitd promising performance, its effectiveness diminished over multiple cycles. Therefore, strategies to regenerate or rejuvenate the adsorbent material between cycles may be necessary to maintain or restore its adsorption capacity^62^. In summary, the results presented in Fig. 9 emphasize the significance of evaluating the reusability of adsorbent materials for lead removal applications. Although the silica/klucel nanocomposite showed effective lead removal initially, its performance declines over successive cycles, highlighting the need for strategies such as regeneration or replacement to ensure sustained efficacy in practical applications.

### Comparative study with other adsorbents

Table 2 displayed a comparative analysis of the adsorption capacities exhibited by different grafted adsorbents. These adsorbents have undergone modifications with distinct chelating groups or grafting agents, resulting in variations in their adsorption performance. The adsorption capacities, expressed in mg/g, provide insights into the effectiveness of each grafted adsorbent in removing contaminants from the solution^63^. For instance, the modification of Klucel with silica leads to an adsorption capacity of 63.9 mg/g, suggesting the potential of this alteration to bolster the adsorption efficiency of Klucel-derived materials.

**Table 2:** Adsorption capacity comparison of the various adsorbents for Pb(II) removal.

Adsorbent	q_max_ (mg/g)	Refs.
Magnetic cellulose nanocomposite beads entrapping activated bentonite	12.6	^64^
SiO_2_@OPW	200	^13^
Starch croos-linked acrylamide and sodium acrylate)	47.11	^61^
Nanosized SiO_2_	32.3	^65^
Chitosan and poly(acrylamide)/Cu nanocomposite	38.93	^60^
Alg_S + SiO_2_	439	^66^
Cellulose grafted dicyclohexyl-18-crown-6	58.3	^3^
Nanohydroxyapatite–alginate	236	^67^
Silica /klucel nanocomposite	63.9	This study

### Potential applications of silica/klucel nanocomposite and future prospects

Silica/klucel nanocomposites hold great promise for environmental remediation due to their unique properties and versatile applications. Silica/klucel nanocomposites possess the capability to efficiently extract heavy metals like lead, cadmium, and arsenic from polluted water reservoirs via adsorption mechanisms. Their high surface area and tailored surface chemistry enable efficient binding of metal ions, leading to clean and safe drinking water. Silica/klucel nanocomposites can be utilized for in-situ soil remediation to degrade organic pollutants like pesticides, herbicides, and industrial chemicals. Functionalized nanocomposites with catalytic properties can facilitate the degradation of contaminants, restoring soil health and fertility. These nanocomposites can be integrated into wastewater treatment setups to eliminate organic pollutants, pharmaceuticals, and personal care products from effluent streams. Advanced oxidation processes involving silica/klucel nanocomposites can efficiently degrade recalcitrant compounds, ensuring compliance with environmental regulations. Silica/klucel nanocomposites can be deployed for the cleanup of oil spills in marine environments. Their high adsorption capacity and affinity for hydrophobic molecules enable the selective removal of oil and hydrocarbons from water surfaces, minimizing environmental damage and protecting marine ecosystems. Silica/klucel nanocomposites can be incorporated into air filtration systems to capture volatile organic compounds emitted from industrial processes, vehicles, and indoor activities. Functionalized nanocomposites can selectively adsorb VOCs, improving indoor and outdoor air quality. These nanocomposites can function as filtration media, effectively capturing particulate matter from industrial exhaust fumes and indoor air. Their porous structure and high surface area enable efficient capture of fine particles, reducing respiratory health risks and environmental pollution. Silica/klucel nanocomposites can be applied in groundwater remediation efforts to treat contaminant plumes arising from industrial spills, landfills, and underground storage tanks. Functionalized nanocomposites can target specific contaminants and facilitate their immobilization or degradation, preventing further groundwater contamination. Prospects for silica/klucel nanocomposites in environmental remediation involve advancements in material synthesis, characterization, and application technologies. Tailoring the properties of nanocomposites to target specific contaminants and environmental conditions will enhance their effectiveness in remediation processes. Moreover, interdisciplinary research collaborations and investment in nanotechnology will drive innovation in the development of next-generation nanocomposites for environmental applications. By harnessing the potential of silica/klucel nanocomposites, we can address pressing environmental challenges and create sustainable solutions for a cleaner and healthier planet.

## Conclusion

The silica/klucel nanocomposite exhibits considerable potential as a proficient and environmentally sound adsorbent for removing lead from industrial wastewater. Its exceptional adsorption capabilities, stability, reusability, and environmental compatibility position it as a promising solution for combating heavy metal pollution in wastewater treatment processes. There is a pressing need for further research and development efforts to refine its synthesis, regeneration processes, and scale-up for practical deployment in industrial settings. The synthesized silica/klucel nanocomposite displayed promising attributes for efficiently removing lead ions from industrial wastewater. Morphological analysis revealed a uniform dispersion of silica nanoparticles within the klucel matrix, imparting a large surface area and mesoporous structure conducive to adsorption. The structural analysis confirmed the successful integration of silica nanoparticles into the klucel matrix, as evidenced by characteristic peaks in the FTIR spectra representing both components. Batch adsorption experiments showcased swift kinetics, reaching equilibrium promptly, and adhering to pseudo-second-order kinetics, indicating chemisorption as the rate-controlling factor. The Langmuir isotherm model exhibited excellent agreement with the experimental data, implying monolayer adsorption of lead ions onto the nanocomposite surface. The adsorption capacity of the nanocomposite was impacted by variables such as solution pH, initial lead ion concentration, temperature, and adsorbent dosage. Evaluation of regeneration methods revealed that acidic and alkaline solutions, along with complexing agents, effectively desorbed the adsorbed lead ions from the nanocomposite surface, with desorption efficiencies surpassing 65%. Reusability testing confirmed the stability and reusability of the nanocomposite over multiple adsorption-regeneration cycles, with minimal loss in adsorption capacity even after seven cycles. Environmental assessments indicated favorable implications of the adsorption process using the silica/klucel nanocomposite, with minimal secondary waste generation and negligible release of adsorbed contaminants. Comparative analysis with other reported adsorbents in the literature showcased competitive adsorption capacity and superior reusability of the nanocomposite. Ultimately, the silica/klucel nanocomposite emerges as a highly promising and eco-friendly adsorbent for mitigating lead contamination in industrial wastewater. Its notable attributes, including high adsorption capacity, swift kinetics, robust stability, and reusability, position it as a feasible solution for wastewater treatment endeavors. To fully harness its potential, further refinements in synthesis protocols, investigation into innovative regeneration methods, and scaling-up endeavors are advocated to bolster its efficacy and applicability in practical scenarios.

## Electronic supplementary material

Below is the link to the electronic supplementary material.

Supplementary Material 1

## Data Availability

Data availability The datasets used and/or analysed during the current study are available from the authors on reasonable request.
