# Supplementary material for: Silica/klucel nanocomposite as promising durable adsorbent for lead removal from industrial effluents
Source: Sci Rep. 2024 Oct 30;14:26095. doi: 10.1038/s41598-024-74680-2 (PMC11525666; doi:10.1038/s41598-024-74680-2)
Supplement: Supplementary file 1 — Supplementary Material 1 [file 41598_2024_74680_MOESM1_ESM.doc]

**2.2.3. Characterization of Silica/Klucel Nanocomposite**

The morphology and microstructure of the nanocomposite was scrutinized through the utilization of scanning electron microscopy SEM. SEM imaging delved into the surface morphology and particle size distribution, offering a comprehensive understanding of the structure and dispersion of silica nanoparticles within the klucel matrix. Meanwhile, Fourier-transform infrared spectroscopy (FTIR) was deployed to dissect the chemical composition and functional groups inherent in the nanocomposite. Spanning the 400-4000 cm^-1^ range, FTIR spectra pinpointed characteristic peaks affiliated with silica, klucel, and any interactions between these constituents. To delve deeper into the specific surface area and pore structure of the nanocomposite, a Brunauer-Emmett-Teller (BET) analysis was enacted. Nitrogen adsorption-desorption isotherms was meticulously measured at a temperature corresponding to liquid nitrogen (-196°C), with subsequent application of the BET equation to compute the surface area and pore volume of the adsorbent [^1^](#_ENREF_1).

**2.2.4. Optimization by Batch Adsorption Experiments**

Prepare a standardized solution containing lead ions (Pb^2+^) with a precisely known concentration by dissolving lead nitrate (Pb(NO₃)₂ meticulously in deionized water. Tailor the pH of the lead solution to the required range, employing either hydrochloric acid (HCl) or sodium hydroxide (NaOH) solutions, and concoct lead solutions at diverse pH levels, encompassing acidic, neutral, and alkaline states. Execute batch adsorption experiments across a spectrum of temperatures, encompassing room temperature as well as 25°C, 35°C, and 45°C, ensuring temperature regulation via a water bath or a temperature-controlled incubator. Vary the quantity of the silica/klucel nanocomposite adsorbent and introduce different doses into the lead solution while holding other parameters constant .Embark on the determination of adsorption kinetics by meticulously monitoring lead removal efficiency at assorted time intervals, such as 15 minutes, 30 minutes, 1 hour and 2 hours maintaining a steady pH, temperature, and adsorbent dose throughout the process .Fabricate lead solutions characterized by differing initial concentrations, ranging from 10 mg/L to 50 mg/L, to evaluate the adsorption capacity of the nanocomposite adsorbent.In each batch adsorption experiment, add a predetermined volume of the lead solution to a series of conical flasks, and add the appropriate amount of the silica/klucel nanocomposite adsorbent to each flask. Stir the solution-adsorbent mixture using a magnetic stirrer or orbital shaker for thorough mixing and allow the adsorption process to proceed for the specified contact time. After the desired adsorption time, separate the adsorbent from the solution using filtration or centrifugation. Analyze the remaining lead concentration in the solution employing advanced analytical methodologies such as atomic absorption spectroscopy (AAS). Plot graphical representations depict lead removal efficiency (%) against variables such as pH, temperature, adsorbent dose, contact time, and initial lead concentration [^2^](#_ENREF_2). Identify the optimal conditions for maximizing lead removal and achieving high adsorption capacity and summarize the findings of the study. Provide recommendations for the practical application of the silica/klucel nanocomposite adsorbent in lead removal from industrial effluents [^3^](#_ENREF_3).

**2.2.5. Adsorption Isotherms**

Adsorption isotherms represent the equilibrium relationship between the concentration of adsorbate molecules on an adsorbent material's surface and the concentration of adsorbate molecules in the bulk phase at a constant temperature. These isotherms offer invaluable insights into adsorption behavior and capacity, facilitating the comprehension and optimization of adsorption processes. Mathematical models have been developed to elucidate adsorption isotherms, with prominent examples including the Langmuir, Freundlich, and BET (Brunauer-Emmett-Teller) models. Each model offers different insights into the adsorption process and is applicable under specific conditions. The Langmuir isotherm posits a scenario of monolayer adsorption on a uniform surface featuring a limited quantity of identical adsorption sites. It implies that once these sites are filled, adsorption proceeds without interaction among adsorbate molecules. The Freundlich isotherm, on the other hand, describes multilayer adsorption onto heterogeneous surfaces with an exponential relationship between adsorbate concentration and adsorption capacity. It indicates that adsorption occurs on surfaces with varied energies and affinities. The BET isotherm is commonly used for physical adsorption onto porous materials and accounts for monolayer and multilayer adsorption. These models offer valuable insights into the adsorbent material's specific surface area and the energies involved in adsorption. Experimental data garnered from adsorption isotherm investigations are matched against these models to ascertain crucial parameters such as maximum adsorption capacity, equilibrium adsorption constant, and adsorption energy. These parameters help in understanding the adsorption mechanism, optimizing adsorption conditions, and predicting the performance of adsorbents in practical applications [^4^](#_ENREF_4).

**2.2.6. Regeneration and Reusability**

Regeneration and reusability are essential aspects of adsorbent materials used in adsorption processes for wastewater treatment and pollutant removal. Regeneration refers to the process of restoring the adsorption capacity of spent adsorbents by removing adsorbed contaminants or restoring their adsorption sites, thereby allowing them to be reused multiple times. Reusability, on the other hand, refers to the ability of adsorbent materials to maintain their adsorption capacity and effectiveness over multiple cycles of adsorption and regeneration. The regeneration process typically involves desorbing the adsorbed contaminants from the surface of the adsorbent material through various methods such as thermal treatment (e.g., heating), chemical regeneration (e.g., elution with desorbing agents), or physical techniques (e.g., vacuum desorption). The selection of a regeneration approach hinges on several factors, including the characteristics of the adsorbate, the mechanism of adsorption, and the attributes of the adsorbent material. Following regeneration, the adsorbent material can be redeployed for subsequent cycles of adsorption. Nonetheless, frequent regeneration and reuse could induce alterations in the adsorbent's adsorption capacity, structural robustness, and surface characteristics, potentially impacting its efficacy over time. Therefore, it is essential to evaluate the reusability of adsorbents through experimental studies, assessing parameters such as adsorption capacity, kinetics, and stability over multiple cycles. Effective regeneration and reusability strategies are crucial for enhancing the sustainability and cost-effectiveness of adsorption processes in wastewater treatment. Extending the lifespan of adsorbent materials and reducing the need for frequent replacement, regeneration, and reusability contribute to the efficient utilization of resources and the minimization of environmental impacts associated with adsorbent disposal [^5^](#_ENREF_5).

**2.2.7. Environmental Implications**

The study assessd the environmental impacts of employing the silica/klucel nanocomposite for adsorption, examining aspects like the production of secondary waste, the discharge of adsorbed contaminants, and the overall ecological suitability. Evaluating both the advantages and potential drawbacks of this adsorption method is crucial to ascertaining its environmental soundness and long-term viability. Through this thorough approach, the research aims to delve into the adsorption capabilities, mechanisms, and environmental ramifications of utilizing the silica/klucel nanocomposite as an effective means of removing lead from industrial wastewater [^6^](#_ENREF_6).

**References**

1 Rajesh Kumar and Rajeev Kr Sharma, "Synthesis and characterization of cellulose based adsorbents for removal of Ni (II), Cu (II) and Pb (II) ions from aqueous solutions," Reactive and Functional Polymers **140**, 82-92 (2019 (

2 Ragab E Abou-Zeid, Kholod H Kamal, ME Abd El-Aziz, SM Morsi, and Samir Kamel, "Grafted TEMPO-oxidized cellulose nanofiber embedded with modified magnetite for effective adsorption of lead ions," International Journal of Biological Macromolecules **167**, 1091-1101 (2021)

3 Okon-Akan Omolabake Abiodun, Oluwasogo Oluwaseun, Olaoye Kayode Oladayo, Omoogun Abayomi, Akpowu Arubi George, Emmanuel Opatola, Robinson Friday Orah, Efe Jeffery Isukuru, Ifunanya Chiamaka Ede, and Oluwadara Temitayo Oluwayomi, "Remediation of heavy metals using biomass-based adsorbents: adsorption kinetics and isotherm models," Clean Technologies **5** (3), 934-960 (2023 .(

4 Imran Rahman Chowdhury, Shakhawat Chowdhury, Mohammad Abu Jafar Mazumder, and Amir Al-Ahmed, "Removal of lead ions (Pb2+) from water and wastewater: a review on the low-cost adsorbents," Applied Water Science **12** (8), 185 (2022) .

5 Yan Chen, Zeguang Nie, Junkai Gao, Jiaqi Wang, and Miaomiao Cai, "A novel adsorbent of bentonite modified chitosan-microcrystalline cellulose aerogel prepared by bidirectional regeneration strategy for Pb (II) removal," Journal of Environmental Chemical Engineering **9** (4), 105755 (2021) .

6 Aminur Rahman, "Promising and environmentally friendly removal of copper, zinc, cadmium, and lead from wastewater using modified shrimp-based chitosan," Water **16** (1), 184 (2024)
